# Systems for grading the quality of evidence and the strength of recommendations I: Critical appraisal of existing approaches The GRADE Working Group

**DOI:** 10.1186/1472-6963-4-38

**Published:** 2004-12-22

**Authors:** David Atkins, Martin Eccles, Signe Flottorp, Gordon H Guyatt, David Henry, Suzanne Hill, Alessandro Liberati, Dianne O'Connell, Andrew D Oxman, Bob Phillips, Holger Schünemann, Tessa Tan-Torres Edejer, Gunn E Vist, John W Williams, The GRADE Working Group

**Affiliations:** 1 Center for Practice and Technology Assessment, Agency for Healthcare Research and Quality, 540 Gaither Rd. Rokville, MD 20852, USA; 2 Centre for Health Services Research, University of Newcastle upon Tyne, 21 Claremont Place, Newcastle upon Tyne NE2 4AA, UK; 3 Informed Choice Research Department, Norwegian Health Services Research Centre, Pb. 7004 St. Olavs Plass, 0130 Oslo, Norway; 4 Departments of Clinical Epidemiology and Biostatistics and Medicine, McMaster University, 1200 Main Street West, Hamilton, Ontario L8N 3Z5, Canada; 5 Department of Clinical Pharmacology, Faculty of Medicine and Health Sciences, University of Newcastle, Level 5, New Med 2 Building, Newcastle Mater Hospital, Waratah, NSW 2298, Australia; 6 Department of Oncology and Hematology, Università di Modena e Reggio Emilia, Azienda Ospedaliera Policlinico, Via dal Pozzo 41, 41100 Modena, Italia and Centro per la Valutazione della Efficacia della Assistenza Sanitaria (CeVEAS), Modena, Italy; 7 Cancer Epidemiology Research Unit, Cancer Research and Registers Division, The Cancer Council NSW, PO Box 572, Kings Cross NSW 1340, Australia; 8 Centre for Evidence-based Medicine, University Department of Psychiatry, Warneford Hospital, Oxford OX3 7JX, UK; 9 Departments of Medicine and Social & Preventive Medicine, University at Buffalo, State University of New York, ECMC-CC142, 462 Grinder St, Buffalo, NY 14215, USA; 10 Global Programme on Evidence for Health Policy, World Health Organisation, CH-1211 Geneva 27, Switzerland; 11 The Center for Health Services Research in Primary Care, HSR&D, Department of Veterans Affairs Medical Center and Duke University Medical Center, 508 Fulton St., Durham, NC 27705, USA

**Keywords:** evidence-based health care, levels of evidence, practice guidelines, strength of recommendation, systematic reviews

## Abstract

**Background:**

A number of approaches have been used to grade levels of evidence and the strength of recommendations. The use of many different approaches detracts from one of the main reasons for having explicit approaches: to concisely characterise and communicate this information so that it can easily be understood and thereby help people make well-informed decisions. Our objective was to critically appraise six prominent systems for grading levels of evidence and the strength of recommendations as a basis for agreeing on characteristics of a common, sensible approach to grading levels of evidence and the strength of recommendations.

**Methods:**

Six prominent systems for grading levels of evidence and strength of recommendations were selected and someone familiar with each system prepared a description of each of these. Twelve assessors independently evaluated each system based on twelve criteria to assess the sensibility of the different approaches. Systems used by 51 organisations were compared with these six approaches.

**Results:**

There was poor agreement about the sensibility of the six systems. Only one of the systems was suitable for all four types of questions we considered (effectiveness, harm, diagnosis and prognosis). None of the systems was considered usable for all of the target groups we considered (professionals, patients and policy makers). The raters found low reproducibility of judgements made using all six systems. Systems used by 51 organisations that sponsor clinical practice guidelines included a number of minor variations of the six systems that we critically appraised.

**Conclusions:**

All of the currently used approaches to grading levels of evidence and the strength of recommendations have important shortcomings.

## Background

In 1979 the Canadian task Force on the Periodic Health Examination published one of the first efforts to explicitly characterise the level of evidence underlying healthcare recommendations and the strength of recommendations [1]. Since then a number of alternative approaches has been proposed and used to classify clinical practice guidelines [2-28].

The original approach used by the Canadian Task Force was based on study design alone, with randomised controlled trials (RCTs) being classified as good (level I) evidence, cohort and case control studies being classified as fair (level II) evidence and expert opinion being classified as poor (level III) evidence. The strength of recommendation was based on the level of evidence with direct correspondence between the two; e.g. a strong recommendation (A) corresponded to there being good evidence. A strength of the original Canadian Task Force approach was that it was simple; the main weakness was that it was too simple. Because of its simplicity, it was easy to understand, apply and present. However, because it was so simple there were many implicit judgements, including judgements about the quality of RCTs, conflicting results of RCTs, and convincing results from non-experimental studies.

For example:

• Should a small, poorly designed RCT be considered level I evidence?

• Should RCTs with conflicting results still be considered level I evidence?

• Should observational studies always be considered level II evidence, regardless of how convincing they are?

The original approach by the Canadian Task Force also did not include explicit judgements about the strength of recommendations, such as how trade-offs between the expected benefits, harms and costs were weighed and taken account of in going from an assessment of how good the evidence is to determining the implications of the results for practice.

The GRADE Working Group is an informal collaboration of people with an interest in addressing shortcomings such as these in systems for grading evidence and recommendations. We describe here a critical appraisal of six prominent systems and the results of the critical appraisal.

## Methods

We selected systems for grading the level of evidence and the strength of recommendations that we considered prominent and that included features not captured by other prominent systems. These were selected based on the experience and knowledge of the authors through informal discussion. A description of the most recent version (as of summer 2000) of each of these systems (Appendix 1 to 6), was prepared by one of the authors familiar with the system, and used in this exercise. The following six systems were appraised: the American College of Chest Physicians (ACCP, [see Additional file 1]) [21], Australian National Health and Medical Research Council (ANHMRC, [see Additional file 2]) [17], Oxford Centre for Evidence-Based Medicine (OCEBM, [see Additional file 3]) [16], Scottish Intercollegiate Guidelines Network (SIGN, [see Additional file 4]) [18], US Preventive Services Task Force (USPSTF, [see Additional file 5]) [22], US Task Force on Community Preventive Services (USTFCPS, [see Additional file 6]) [25].

These descriptions of the systems were given to the twelve people who independently appraised the six systems, all of the authors minus GEV appraised the six systems, three of the authors (DH, SH and DO'C) appraised as a group and reported as one (see contributions). The 12 assessors all had experience with at least one system and most had helped to develop one of the six included systems. Twelve criteria described by Feinstein [29] provided the basis for assessing the sensibility of the six systems.

### Criteria used to assess the sensibility of systems for grading evidence and recommendations

1. To what extent is the approach applicable to different types of questions? -effectiveness, harm, diagnosis and prognosis (No, Not sure, Yes)

2. To what extent can the system be used with different audiences? -patients, professionals and policy makers (Little extent, Some extent, Large extent)

3. How clear and simple is the system? (Not very clear, Somewhat clear, Very clear)

4. How often will information not usually available be necessary? (Often, Sometimes, Seldom)

5. To what extent are subjective decisions needed? (Often, Sometimes, Seldom)

6. Are dimensions included that are not within the construct (level of evidence or strength of recommendation)? (Yes, Partially, No)

7. Are there important dimensions that should have been included and are not? (No, Partially, Yes)

8. Is the way in which the included dimensions are aggregated clear and simple? (No, Partially, Yes)

9. Is the way in which the included dimensions are aggregated appropriate? (No. Partially, Yes)

10. Are the categories sufficient to discriminate between different levels of evidence and strengths of recommendations? (No, Partially, Yes)

11. How likely is the system to be successful in discriminating between high and low levels of evidence or strong and weak recommendations? (Not very likely, Somewhat likely, Highly likely)

12. Are assessments reproducible? (Probably not, Not sure, Probably)

No training was provided and we did not discuss the 12 criteria prior to applying them to the six systems.

Our independent appraisal of the six systems were summarised and discussed. The discussion focused on differences in the interpretation of the criteria, disagreement about the judgements that we made and sources of these disagreements, the strengths and weaknesses of the six systems, and inferences based on the appraisals and subsequent discussion.

In order to identify important systems that we might have overlooked following our appraisal of these six systems we also searched the US Agency for Health Care Research and Quality (AHRQ) National Guidelines Clearing House for organisations that have graded two or more guidelines in the Clearing House using an explicit system [30]. These systems were compared with the six systems that we critically appraised.

## Results

There was poor agreement among the 12 assessors who independently assessed the six systems. A summary of the assessments of the sensibility of the six approaches to rating levels of evidence and strength of recommendation is shown in Table 1.

## Discussion

The poor agreement among the assessors likely reflects several factors. Some of us had practical experience using one of the systems or used additional background information related to one or more grading systems, and we may have been biased in favour of the system with which we were most familiar. Each criterion was applied to grading both evidence and recommendations. Some systems were better for one of these constructs than the other and we may have handled these discrepancies differently. In addition each criterion may have been assessed relative to different judgements about the evidence, such as an assessment of the overall quality of evidence for an important outcome (across studies) versus the quality of an individual study. Some of the criteria were not clear and were interpreted or applied inconsistently. For example, a system might be clear and not simple or visa versa. We likely differed in how stringently we applied the criteria. Finally, there was true disagreement.

There was agreement that the OCEBM system works well for all four types of questions. There was disagreement about the extent to which the other systems work well for questions other than effectiveness. It was noted that some systems are not intended to address other types of questions and it is not clear that it is important that a system should address all four types of questions that we considered (effectiveness, harm, diagnosis, prognosis), although criteria for assessing individual studies must take this into account [31,32].

Most of us did not find that any of the systems are likely to be suitable for use by patients. Almost all agreed that the ACCP system was suitable for professionals and most considered that the USPSTF system was suitable for professionals. There was not much agreement about the suitability of any of the other systems for professionals or about the suitability of any of the systems for policy makers, although most assessed the USTFCPS system to be suitable for policy makers.

There was no agreement that any of the systems are clear and simple, although USPSTF, ACCP and SIGN systems were generally assessed more favourably in this regard. It was generally agreed that the clearer a system was the less simple it was; e.g. the OCEBM system is clear but not simple for categorising the level of evidence. There was some confusion regarding whether we were assessing how clear and simple the system was to guideline developers (as some interpreted this criterion) or how clear and simple the outcome of applying the system was to guideline users (as others interpreted this criterion). Either way, the simpler a system is the less clear it is likely to be.

Most of us judged that for most of the systems necessary information would not be available at least sometimes. The OCEBM system came out somewhat better than the other systems and lack of availability of necessary information was considered to be less of a problem for the USTFCPS system. However, the OCEBM and USTFCPS systems were considered by most to be missing dimensions which may, in part, explain why missing information was considered to be less of a problem. This would be the case to the extent the missing dimensions were the ones for which information would often or sometimes not be available. The dimension for which we considered that information would most often be missing was trade-offs; i.e. knowledge of the preferences or utility values of those affected. Additional problems were identified in relationship to complex interventions and counselling, particularly with the USTFCPS and USPSTF systems. It was pointed out that the USTFCPS system addressed this problem by including availability of information about the intervention as part of its assessment of the quality of evidence.

Most of the systems were assessed to require subjective decisions at least to some extent. The OCEBM system again stood out as being assessed more favourably, although it may be related to omission of dimensions that require more subjective decisions. Judgement is clearly needed with any system. The aim should be to make judgements transparent and to try to protect against bias in the judgements that are made by being systematic and explicit.

Inclusion of dimensions that are not within the constructs being graded was not considered a problem for most of the systems by most of us. Several people considered that it might be a problem for the USTFCPS and USPSTF systems. On the other hand, all of the systems were evaluated to be missing at least one important dimension by at least one person. The challenge of missing dimensions were considered less of a problem for the ACCP and ANHMRC systems. There was not agreement about any of the systems having a clear and simple approach to aggregating the dimensions, although this was considered to be less of a problem for the ACCP, SIGN and USTFCPS systems.

There was also not agreement on the appropriateness of how the dimensions were aggregated. This was considered to be more of a problem for the ANHMRC and USTFCPS systems than the other four systems, all of which were considered to have taken an approach to aggregating the dimensions that was at least partially inappropriate by more than half of us.

Most of us considered that most of the systems had sufficient categories, with the exception of the ANHMRC system. There was almost agreement that the USPSTF system has sufficient categories. We agreed that it is possible to have too many categories as well as too few, the OCEBM system being an example of having too many categories.

There was not agreement that any of the systems are likely to discriminate successfully, although everyone thought that the ACCP, SIGN and USPSTF systems are somewhat to highly likely to discriminate. Lastly, we largely agreed that we were not sure how reproducible assessments are using any of the systems, although half of us considered that assessments using the ANHMRC system are unlikely to be reproducible and about 1/3 considered that assessments using the OCEBM and ACCP systems are likely to be reproducible.

We identified 22 additional organisations that have produced 10 or more practice guidelines using an explicit approach to grade the level of evidence or strength of recommendations. Another 29 have produced between two and nine guidelines using an explicit approach. These systems include a number of minor variations of the six systems that we appraised in detail.

There was generally poor agreement between the individual assessors about the scoring of the six approaches using the 12 criteria. However, there was general agreement that none of these six prominent approaches to grading the levels of evidence and strength of recommendations adequately addressed all of the important concepts and dimensions that we thought should be considered. Although we limited our appraisal to six systems all of the additional approaches to grading levels of evidence and strength of recommendations that we identified were, in essence, variations of the six approaches that we had critically appraised. Therefore we are confident that we did not miss any important grading systems available at the time when these assessments were undertaken.

Based on discussions following the critical appraisal of these six approaches, we agreed on some conclusions:

• Separate assessments should be presented for judgements about the quality of the evidence and judgements about the balance of benefits and harms.

• Evidence for harms should be assessed in the same way as evidence for benefits, although different evidence may be considered relevant for harms than for benefits; e.g. local evidence of complication rates may be considered more relevant than evidence of complication rates from trials for endarterectomy.

• Judgements about the quality of evidence should be based on a systematic review of the relevant research.

• Systematic reviews should not be included in a hierarchy of evidence (i.e. as a level or category of evidence). The availability of a well-done systematic review does not correspond to high quality evidence, since a well-done review might include anything from no studies to poor quality studies with inconsistent results to high quality studies with consistent results.

• Baseline risk should be taken into consideration in defining the population to whom a recommendation applies. Baseline risk should also be used transparently in making judgements about the balance of benefits and harms. When a recommendation varies in relationship to baseline risk, the evidence for determining baseline risk should be assessed appropriately and explicitly.

• Recommendations should not vary in relationship to baseline risk if there is not adequate evidence to guide reliable determinations of baseline risk.

## Conclusions

Based on discussions of the strengths and limitations of current approaches to grading levels of evidence and the strength of recommendations, we agreed to develop an approach that addresses the major limitations that we identified. The approach that the GRADE Working Group has developed is based on the discussions following the critical appraisal reported here and a pilot study of the GRADE approach [33]. Based on the pilot testing and the discussions following the pilot, the GRADE Working Group has further developed the GRADE system to its present format [34].

The GRADE Working Group has continued to grow as an informal collaboration that meets one or two times per year. The group maintains web pages  and a discussion list.

## Competing interests

DA has competing interests with the US Preventive Services Task Force (USPSTF), PAB has a competing interest with the US Task Force on Community Preventive Services (USTFCPS), GHG and HS have competing interests with the American College of Chest Physicians (ACCP), DH, SH and DO'C have competing interests with the Australian National Health and Medical Research Council (ANHMRC), BP has competing interests with the Oxford Centre for Evidence-Based Medicine (OCEBM). Most of the other members of the GRADE Working Group have experience with the use of one or more systems of grading evidence and recommendations.

## Contributions

DA, PAB, ME, SF, GHG, DH, SH, AL, DO'C, ADO, BP, HS, TTTE, GEV & JWW Jr as members of the GRADE Working Group have contributed to the preparation of this manuscript and the development of the ideas contained herein, participated in the critical assessment, and read and commented on drafts of this article. GHG and ADO have led the process. GEV has had primary responsibility for coordinating the process.

## Pre-publication history

The pre-publication history for this paper can be accessed here:



## Supplementary Material

Additional File 1 American College of Chest Physicians (ACCP), a brief description of the ACCP approach. Click here for file

Additional File 2 Australian National Health and Medical Research Council (ANHMRC), a brief description of the ANHMRC approach. Click here for file

Additional File 3 Oxford Centre for Evidence-based Medicine (OCEBM), a brief description of the OCEBM approach. Click here for file

Additional File 4 Scottish Intercollegiate Guidelines (SIGN), a brief description of the SIGN approach. Click here for file

Additional File 5 U.S. Preventive Services Task Force (USPSTF), a brief description of the USPSTF approach. Click here for file

Additional File 6 U.S. Task Force on Community Preventive Services (USTFCPS), a brief description of the USTFCPS approach. Click here for file

## Figures and Tables

**Table 1:** Summary of assessments of the sensibility of six approaches to rating levels of evidence and strength of recommendation

Criteria^1^	ACCP			ANHMRC^2^			USTFCPS			OCEBM			SIGN			USPSTF^3^		
	No		Yes	No		Yes	No		Yes	No		Yes	No		Yes	No		Yes
1. Applicable to different questions:																		
Effectiveness			12		2	8		1	11			12	1		11		2	9
Harm		1	11		5	5	1	7	4		1	11	1	3	8	2	2	7
Diagnosis	7	3	2	4	4	2	9	3				12	5	2	5	2	2	7
Prognosis	6	3	3	2	5	3	9	2	1			11	4	3	5	3	3	5
																		
2. Can be used by:																		
Professionals		1	11	1	5	3		7	4	1	6	5		5	7		3	8
Policy makers	1	5	6	1	5	3	1	2	9	3	7	2	2	6	4	1	4	6
Patients	4	5	3	5	5		6	3	3	9	3		7	5		4	6	1
																		
3. Clear and simple	1	5	6	2	6	1	2	8	2	2	4	5	1	5	6	1	4	7
4. Information not available		8	4	1	5	3	1	6	5		4	8	1	7	4	1	9	2
5. Subjective decisions	2	10		5	2	2	5	5	2		7	5	5	7		2	9	
6. Inappropriate dimensions	1	3	8		1	6	2	4	6		1	10	1	2	8	1	4	6
7. Missing dimensions	1	6	5	2	2	4	5	4	3	9	3	1	5	4	3	2	5	4
																		
Aggregation of dimensions:																		
8. Clear and simple	1	5	6	4	1	2	2	2	7	3	4	4		6	6	2	7	2
9. Appropriate		6	5	3	1	1	3	4	4	2	5	4	1	4	6	1	5	5
																		
10. Sufficient categories	1	4	6	4	2	1		5	7	2	2	7	1	2	9		1	10
11. Likely to discriminate		7	5	2	5	1	1	9	2	2	4	6		5	7		4	7
12. Assessments reproducible	1	8	3	4	4		2	7	2		7	4	1	8	2		10	

^1^See Criteria described in Methods.

^2^Two people did not assess the ANHMRC because it was more descriptive and others responded not applicable for some questions.

^3^One person did not assess the USPST and one person had two responses on questions 3 and 4.
