# Supplementary material for: Systems for grading the quality of evidence and the strength of recommendations I: Critical appraisal of existing approaches The GRADE Working Group
Source: BMC Health Serv Res. 2004 Dec 22;4:38. doi: 10.1186/1472-6963-4-38 (PMC545647; doi:10.1186/1472-6963-4-38)
Supplement: Additional File 1 — American College of Chest Physicians (ACCP), a brief description of the ACCP approach. [file 1472-6963-4-38-S1.doc]

**APPENDIX 1.**

**American College of Chest Physicians (ACCP)**

Brief description prepared by Holger Schünemann.

# Background

The Consensus Conferences on Antithrombotic Therapy of the American College of Chest Physicians (ACCP) has developed guidelines to help clinicians make antithrombotic treatment decisions in average patients. Experts reviewed a broad spectrum of indications for antithrombotic therapy across different conditions and indications, such as pregnancy, in children, after surgery and in coronary artery disease. As part of the first consensus conference in 1986, Dave Sackett suggested a formal rating scheme for assessing levels of evidence derived from the Canadian Taskforce on the Periodic Health Examination [1-3]. Gordon Guyatt and Deborah Cook have lead the evolution of these “rules of evidence” [4-10], which the experts have applied to generate grades of recommendations during the subsequent meetings.

The last revision of this approach that focuses on therapy, harm and to a smaller extent on diagnosis was published in January 2001 [10]. It provides a clearer separation of bias and random error than initial versions. This approach is widely known, because of the substantial distribution and publication of the proceedings from this ACCP Consensus Conference (200,000 copies of a journal supplement and a pocket guide, respectively, have been published in 2001) [11, 12].

# Quality of evidence

In the current ACCP grading system assignment of Grades A to C focuses on the likelihood of bias and is based on the methodological quality of the underlying evidence. Randomized controlled trials (RCTs) with consistent results are classified as **Grade A**. Randomized trials with inconsistent results, or with major methodological weaknesses are assigned **Grade B**. Evidence leading to **Grade C** comes from observational studies and from generalization from randomized trials in one group of patients to a different group. When experts find the generalization from randomized trials secure or the data from observational studies overwhelmingly compelling, they choose **Grade C+**. For example, generalizing the results of oral anticoagulation therapy from RCTs in patients with atrial fibrillation without mitral valve disease (**Grade A** for methodological quality) to patients who have atrial fibrillation with mitral valve disease generated **Grade C**+ in terms of methodological quality for the latter. This generalization is secure because in patients with mitral valve disease and atrial fibrillation the risk of embolism is high and the biology of embolism as well as warfarin action are very similar (Table 1).

**Strength of recommendations**

The ACCP approach aims at expressing the quality of the evidence as part of the strength of recommendation and, thus, the quality of evidence contributes directly to the grade of the recommendation. The magnitude of random error is captured in the decision about our confidence in the trade-off between benefits and risks. The uncertainty associated with this trade-off will determine the strength of recommendations. If experts are very certain that benefits do, or do not, outweigh risks, they will make a strong recommendation -- in our formulation, **Grade 1**. If they are less certain of the magnitude of the benefits and risks, and thus their relative impact, they must make a weaker **Grade 2** recommendation. The approach expresses the primacy of the risk/benefit judgement in determining the recommendation and its strength by placing it first. The grades generated are 1A, 1B, 1C+, 1C, 2A, 2B and 2C, shown in Table 1.

# Strengths and weaknesses

There are several advantages to the ACCP approach. First, the ACCP approach has evolved over 15 years. Methodologists and sophisticated expert clinicians have subjected the approach to intense scrutiny, and the scrutiny has resulted in repeated improvements to the formulation. Second, the approach is relatively simple. In particular, clinicians can focus on the numeric grade, and see either a strong or weak recommendation. This two category approach has a clear clinical correlate: the clinician can apply strong clinical recommendations to most patients without hesitation, while careful thought and discussion with the patient are likely to be required for weak recommendations. Third, the linking of methodological strength with the grade of recommendation reminds clinicians of the importance of considering the strength of evidence in formulating recommendations, and in making clinical decisions. Fourth, clinicians have become familiar with this approach because the widespread distribution of the ACCP Antithrombotic Therapy Guidelines.

Since clinicians do not make recommendations for prognosis the assessment of the quality of evidence for studies evaluating disease prognosis is not practicable with this approach. Other criticisms have been that guidelines in areas of health care and public health that lack evidence from clinical trials would reveal uniformly **Grade C** or **Grade** **C+** recommendations and generating the latter grade could include subjective decisions. Although there is little reason to believe that this approach could not be applied to guidelines and recommendations in other areas of health care, previous versions of this approach have been used little outside the antithrombotic therapy area.

# Target audiences

The target audiences are clinicians providing therapy, including trainees in internal medicine, general practitioners, specialists and sub-specialists.

# Guidelines made with the use of this approach

The ACCP Approach in its current, recently updated form has been applied to guidelines from the Sixth ACCP Consensus Conference on Antithrombotic Therapy (<http://www.chestjournal.org/content/vol119/1_suppl/>,^11^ <http://www.chestnet.org/health.science.policy/quick.reference.guides/antithrombotic/index.html>.^12^ These guidelines entail several hundred specific recommendations across different conditions and target groups that require antithrombotic therapy.

# Evaluations of this approach

There are no formal evaluations completed to date. We plan to measure agreement in grading recommendations between clinician experts and methodologists using data from the upcoming Seventh ACCP Consensus Conference on Antithrombotic Therapy.

Consensus conference participants have provided feedback on the applicability of the approach during each of the consensus conferences. Methodologists have provided informal feedback on strengths and weaknesses of this approach. There is informal feedback from clinician experts, users and trainees.

**Table 1: Current Approach to Grades of Recommendations**

| **Grade of recommendation** | **Clarity of risk/benefit** | **Methodologic strength of supporting evidence** | **Implications** | **Example of recommendation** |
| --- | --- | --- | --- | --- |
| **1A** | Risk/benefit clear | Randomized controlled trials (RCTs) without important limitations | Strong recommendation, can apply to most patients in most circumstances without reservation | We recommend warfarin therapy in patients with atrial fibrillation at high risk for stroke |
| **1 B** | Risk/benefit clear | RCTs with important limitations (inconsistent results, methodological flaws*) | Strong recommendations, likely to apply to most patients | We recommend that pentoxifylline should **not** be used in patients with intermittent claudication |
| **1 C+** | Risk/benefit clear | No RCTs but RCT results can be unequivocally extrapolated, or overwhelming evidence from observational studies | Strong recommendation, can apply to most patients in most circumstances | We recommend long-term warfarin therapy for patients with atrial fibrillation **and** rheumatic mitral valve disease |
| **1 C** | Risk/benefit clear | Observational studies | Intermediate strength recommendation; may change when stronger evidence available | We recommend surveillance and postpartum anticoagulation for pregnant patients with prior venous thromboembolism associated with a transient risk factor |
| **2A** | Risk/benefit unclear | RCTs without important limitations | Intermediate strength recommendation, best action may differ depending on circumstances or patients’ or societal values | We do **not** recommend aspirin as sole therapy in patients after hip fracture surgery to prevent venous thromboembolism |
| **2 B** | Risk/benefit unclear | RCTs with important limitations (inconsistent results, methodological flaws) | Weak recommendation, alternative approaches likely to be better for some patients under some circumstances | We recommend early anticoagulation in patients with acute cardioembolic large-artery ischemic stroke who are ineligible for thrombolysis |
| **2 C** | Risk/benefit unclear | Observational studies | Very weak recommendations; other alternatives may be equally reasonable | We recommend long term aspirin therapy in patients with bioprosthetic heart valves who are in normal sinus rhythm |

* These situations include RCTs with both lack of blinding and subjective outcomes, where the risk of bias in measurement of outcomes is high, RCTs with large loss to follow up.

NOTE: Since studies in categories B and C are flawed, it is likely that most recommendations in these classes will be grade 2.

The following considerations will bear on whether the recommendation is Grade 1 or 2: the magnitude and precision of the treatment effect, patients’ risk of the target event being prevented, the nature of the benefit, and the magnitude of the risk associated with treatment, variability in patient preferences, variability in regional resource availability and health care delivery practices, and cost considerations. Inevitably, weighing these considerations involves subjective judgement.

**References**

1. The periodic health examination. Canadian Task Force on the Periodic Health Examination. CMAJ 1979;121(9):1193-254.
2. Sackett DL. Rules of evidence and clinical recommendations on the use of antithrombotic agents. Chest 1986;89(2 Suppl):2S-3S.
3. Sackett DL. Rules of evidence and clinical recommendations on the use of antithrombotic agents. Archives Int Med 1986:146:464-465.
4. Guyatt GH, Sackett DL, Sinclair JC, Hayward R, Cook DJ, Cook RJ. Users' guides to the medical literature. IX. A method for grading health care recommendations. Evidence-Based Medicine Working Group. JAMA 1995;274(22):1800-4.
5. Sackett DL. Rules of evidence and clinical recommendations on the use of antithrombotic agents. Chest 1989;95:2S-4S.
6. Cook DJ, Guyatt GH, Laupacis A, Sackett DL. Rules of evidence and clinical recommendations on the use of antithrombotic agents. Chest 1992;102:305S-11S.
7. Cook DJ, Guyatt GH, Laupacis A, Sackett DL, Goldberg RJ. Clinical recommendations using levels of evidence for antithrombotic agents. Chest 1995;108:227S-30S.
8. Guyatt GH, Sackett DL, Sinclair J, Hayward RS, Cook DJ, Cook RJ. Users' Guides to the Medical Literature. IX. A Method for Grading Health Care Recommendations. JAMA. 1995;274:1800-1804
9. Guyatt GH, Cook DJ, Sackett DL, Eckman M, Pauker S. Grades of recommendation for antithrombotic agents. Chest 1998;114(5 Suppl):441S-4S. http://www.chestjournal.org/content/vol119/1_suppl/
10. Guyatt G, Schünemann H, Cook D, Pauker S, Sinclair J, Bucher H, Jaeschke R. Grades of Recommendation for Antithrombotic Agents. Chest 2001;119: 3S-7S.
11. J. Dalen, J. Hirsh, G. Guyatt, editors. Sixth ACCP Consensus Conference on Antithrombotic Therapy. Chest 2001; 119;Supplement:s1-S370.

<http://www.chestjournal.org/content/vol119/1_suppl/>

1. Quick Reference Guide for Clinicians. Sixth ACCP Consensus Conference on Antithrombotic Therapy. Conference Chairs: Dalen, J. Hirsh, G. Guyatt. Editor: H. Schünemann. ACCP, Northbrook, IL, 2001.

<http://www.chestnet.org/health.science.policy/quick.reference.guides/antithrombotic/index.html>
