# Supplementary material for: Systems for grading the quality of evidence and the strength of recommendations I: Critical appraisal of existing approaches The GRADE Working Group
Source: BMC Health Serv Res. 2004 Dec 22;4:38. doi: 10.1186/1472-6963-4-38 (PMC545647; doi:10.1186/1472-6963-4-38)
Supplement: Additional File 2 — Australian National Health and Medical Research Council (ANHMRC), a brief description of the ANHMRC approach. [file 1472-6963-4-38-S2.doc]

APPENDIX 2.

**Australian National Health and Medical Research Council (ANHMRC)**

Brief description prepared by Dianne O’Connell.

**Background**

The Australian National Health and Medical Research Council (ANHMRC) recommends that three questions be asked of a body of data on the efficacy of a medical intervention [1]:

1. Is there a real effect?
2. Is the size of the effect clinically important?
3. Was a relevant outcome measure used?

The first question relates to whether the treatment effect could be due to bias (ie the level and quality of the evidence) or chance (the statistical precision or *P*-value). The second question focuses on the clinical (as opposed to statistical) significance of any treatment effect, and the third concerns the importance to patients of the outcome including the validity of any surrogate outcomes and duration of follow-up. These terms are defined in Table 1.

Table 1. Evidence dimensions - definitions

| Type of evidence (dimension) | Definition |
| --- | --- |
| **Strength of evidence**  Level | The study design used, as an indicator of the degree to which bias has been eliminated by design. |
| Quality | The methods used by investigators to minimise bias within a study design. |
| Statistical precision | The *P*-value or, alternatively, the precision of the estimate of the effect (as indicated by the confidence interval). It reflects the degree of certainty about the existence of a true effect. |
| **Size of effect** | The distance of the study estimate from the ‘null’ value and the inclusion of only clinically important effects in the confidence interval. |
| **Relevance of evidence** | The usefulness of the evidence in clinical practice, particularly the appropriateness of the outcome measures used. |

**Dimensions of evidence**

The *level* of evidence indicates the study design used by the investigators to assess the effectiveness of an intervention. The level assigned to a study reflects the degree to which bias has been eliminated by the study design. The levels are defined in Table 2. In addition to the level of evidence, the quality of the individual studies should be considered. The *quality* of the evidence refers to the methods used by the investigators during the study to minimise bias and control confounding within a study type (ie how well the investigators conducted the study). The important sources of bias, and their possible effects, depend on the type of study. Standard quality assessment instruments for each study type have been developed. The third component in assessing the strength of the evidence is the *statistical precision*. The magnitude of the *P*-value (the false positive rate) and the precision (or width of the confidence interval) of the estimate of the treatment effect are important when assessing the strength of the evidence.

The *size* of the treatment effect refers to the size (or the distance from the null value) of the measure (or point estimate) of treatment effect and the values included in the corresponding 95% CI. In the case of a systematic review, it is the summary measure of effect based on the studies included in the review.

A very important dimension (perhaps the most important) is the *relevance* of the evidence. The focus is on the appropriateness of the outcomes. Are they of importance or of interest to the patient? Are they short-term or long-term effects? How well do they relate, in a causal sense, to outcomes of importance to the patient? Relevance also relates to the extent to which the intervention can be replicated in other settings and the applicability of the study findings to other settings and patient groups unlike those in which its efficacy has been tested.

Table 2. Designation of levels of evidence (Australian NHMRC 199X)

| Level of evidence | Study design |
| --- | --- |
| I | Evidence obtained from a systematic review of all relevant randomised controlled trials. |
| II | Evidence obtained from at least one properly-designed randomised controlled trial. |
| III-1 | Evidence obtained from well-designed pseudorandomised controlled trials (alternate allocation or some other method). |
| III-2 | Evidence obtained from comparative studies (including systematic reviews of such studies) with concurrent controls and allocation not randomised, cohort studies, case-control studies, or interrupted time series with a control group. |
| III-3 | Evidence obtained from comparative studies with historical control, two or more single arm studies, or interrupted time series without a parallel control group. |
| IV | Evidence obtained from case series, either post-test or pretest/post-test. |

Source: NHMRC 1999 [1]

**Strength of recommendations**

The ANHMRC defines ‘strength of evidence’ as in Table 1. However, the guidelines argue against basing ‘strength of recommendation’ on this alone. The guidelines also recommend against reducing evidence to a single metric that represents ‘strength of recommendation’. Instead it is argued that the various dimensions of evidence in Table 1 should be considered in disaggregated form.

It is recommended that the relative importance of the dimensions should be considered in the context of the clinical problem being addressed. For example, evidence from a good quality RCT may be of limited relevance (due to the sub-optimal outcomes measured). In that case the most important basis for a recommendation may be a study from a lower *level* of evidence that provides a precise estimate of a sizeable effect measured as a change in a highly *relevant* outcome measure. An example in recent years has been the avoidance of serious adverse effects of traditional non-steroidal anti-inflammatory drugs (advice based on case-control and cohort studies rather than randomised trials).

A checklist that summarises the data and classifies it according to its *level, quality, statistical precision, relevance* and *the size* of the treatment effect should accompany each major recommendation. This checklist should reflect the results, where possible, from a formal synthesis of the available evidence. If there is no systematic review of the relevant studies, the data from the best available studies should be rated. As mentioned earlier a single strength of recommendation rating using A, B, C etc is not advocated in this process.

**Strengths and weaknesses of the ANHMRC approach**

The advantage of assessing and presenting the evidence in this way is that decision makers can make up their own minds about the intervention based on the dimensions that appear important to the relevant constituencies. Decision-makers can apply specific weights to a particular dimension that reflect the context in which a decision is being made, ranging from a clinical practice guideline for individual patient care through to a policy decision regarding subsidisation of a medical intervention that may involve expenditure of hundreds of millions of dollars. Combining the dimensions into a single “strength of recommendation” cannot address all viewpoints and preferences.

The main weaknes of this method (to some) is that it does not provide a single classification signifying the ‘strength of recommendation’. Another weakness is that it does not consider fully issues of applicability of results to individual patients. These are covered in a separate guide [2]. Finally, the approach does not integrate benefits harms and costs. The dimensions of evidence on each have to be assessed before they are brought together prior to a decision being made.

**Target audiences**

This approach was developed for multidisciplinary groups that are preparing clinical practice guidelines under the auspices of the NHMRC. It is also of value to policy makers who require a summary of a body of data.

**Guidelines**

Groups preparing clinical practice guidelines are using the toolkits and this approach currently. Some of the dimensions of evidence have been included in a guide for decision making [3].

# References

1. National Health and Medical Research Council. A guide to the development, implementation and evaluation of clinical practice guidelines. Commonwealth of Australia 1999. <http://www.health.gov.au/nhmrc/publicat/synopses/cp30syn.htm>
2. National Health and Medical Research Council. How to use the evidence: assessment and application of scientific evidence. Commonwealth of Australia 2000. <http://www.health.gov.au/nhmrc/publicat/pdf/cp69.pdf>
3. Craig JC, Irwig LM, Stockler MR. Evidence-based medicine: useful tools for decision making. MJA 2001;174:248-53.
