# Supplementary material for: Systems for grading the quality of evidence and the strength of recommendations I: Critical appraisal of existing approaches The GRADE Working Group
Source: BMC Health Serv Res. 2004 Dec 22;4:38. doi: 10.1186/1472-6963-4-38 (PMC545647; doi:10.1186/1472-6963-4-38)
Supplement: Additional File 3 — Oxford Centre for Evidence-based Medicine (OCEBM), a brief description of the OCEBM approach. [file 1472-6963-4-38-S3.doc]

**APPENDIX 3.**

**Oxford Centre for Evidence-based Medicine (OCEBM)**

Brief description prepared by Bob Phillips.

**Background**

The Oxford Centre for Evidence-based Medicine (OCEBM) Levels of Evidence and Grades of Recommendation 1999 [1] were developed in response to a need for assessment of evidence beyond therapeutic interventions. They are an evolution of the Canadian Task Force on the Periodic Health Examination grading system of 1979. The development of the Oxford Levels of Evidence was in response to the writing of a series of guidelines for junior medical staff, the "Evidence-based On Call" project. They cover many aspects of the medical management of patients, including causation and diagnosis as well as therapeutic interventions.

**Quality of evidence**

The *levels of evidence* are derived from a matrix which has four axes, corresponding to the broad type of clinical question under consideration. These are "interventions/aetiology", "prognosis", "diagnosis" and "economic analysis". Each of these axes is divided into 5 broad levels of evidence, ranked from 1 (least potential bias) to 5 (most potential bias). The *level* allocation is primarily dependent on study design factors (e.g. randomisation in interventions, or independent reference standards for diagnosis). Other factors include outcome assessment (e.g. 'minus' when a result is too imprecise) and clinical sensibility (e.g. 'appropriate spectrum' of patients in diagnostic tests). See <http://cebm.jr2.ox.ac.uk/docs/levels.htm>

**Strength of recommendations**

The *grade of recommendation* is a compression of the 10 'levels' into 4 'grades', without any added deliberation or assessment. Level 1a to 1c studies give grade A recommendations; 2a to 3b map to grade B; level 4 studies are grade C and level 5 or imprecise ('minus' level) studies give a grade D recommendation.

**Strengths and weaknesses**

The strengths of the OCEBM approach are in the detailed development of the *levels* of evidence. The different axes allow for questions related to diagnosis, aetiology and prognosis to be considered as 'evidence-based' as well as traditionally intervention-orientated recommendations. Another strength is in the partial incorporation of aspects of heterogeneity into the grade of recommendation. The detailed description of the study levels, and their objectivity, make reproducibility likely to be high. However, this detail may introduce problems for inexperienced users. A study estimating inter-tester reliability has been performed in the Oxford CEBM, and is under analysis (Personal Communication: RSP).

The weakness of the OCEBM approach can be summarised as the simplistic translation of *level* of evidence into *grade* of recommendation. No assessment is made of the clinical importance of the outcomes under consideration. There is no way of balancing of benefits or harms, nor assessment of applicability of the studies. There is no clear way of compiling the body of evidence (often of separate levels) into a single grade of recommendation, or differentiation of direct or indirect evidence.

**Target audiences**

The OCEBM levels of evidence and grades of recommendation are intended to be used by clinicians in practice. This approach is not intended for use by consumers or policy makers.

**Guidelines made with the use of this approach**

The OCEBM approach has been used most extensively by "Evidence-based On Call" to produce 37 guidelines in general (internal) acute medicine [2,3]. This project develops guidelines which are focussed currently on the needs of the postgraduate trainee clinician. The process is of systematic search of the literature, critical abstraction, explicit allocation of a *level of evidence* and summary into a guideline, with each statement given a summary *grade of recommendation*. All aspects of management, from initial presentation, diagnosis, investigation, treatment and prognostication are included in the guides.

The "Evidence-based On Call" internet system has recently been adopted by the UK National Health Service National *electronic* Library of Health (NeLH) [4]. An evaluation of user feedback and utilisation is planned.

Within the field of the project (guidelines in general acute medicine), the homogeneity of the clinical environment and the secondary or tertiary nature of most evidence used, ironed out some of the possible problems. Using the OCEBM approach at a different level in the health care system (e.g. primary care, where different populations are cared for) or across disciplines (e.g. with physiotherapists, when different training and structures are present) may be difficult. We are not aware of any group which has used the OCEBM grading system outside hospital medical practice.

**Studies evaluating the application of guidelines made with this approach**

Formal evaluations completed:

None to date.

Formal evaluations underway or planned:

The NeLH evaluation may include aspects of audit against selected "Evidence-based On Call" guidelines.

Informal evaluations:

Focus groups used during the development of the 'Evidence-based On Call' project demonstrated a desire for such information. A number of clinicians working with the developers of the "Evidence-based On Call" guidelines believed their practice had been altered by the information presented.

**References**

1. <http://cebm.jr2.ox.ac.uk/docs/levels.html> - top
2. [http://www.eboncall.co.uk](http://www.eboncall.co.uk/)
3. Ball, CM & Phillips, RS [Eds.] Evidence-based On Call; Acute Medicine. Harcourt Brace 2001
4. [http://www.nelh.nhs.uk](http://www.nelh.nhs.uk/)

Levels of Evidence and Grades of Recommendations - 23 November 1999.

| Grade of Recommendation | Level of Evidence | Therapy/Prevention, Aetiology/Harm | Prognosis | Diagnosis | Economic analysis |
| --- | --- | --- | --- | --- | --- |
|  | 1a | SR (with homogeneity^i^) of RCTs | SR (with homogeneity*) of inception cohort studies; or a CPG^ii^ validated on a test set. | SR (with homogeneity*) of Level 1 diagnostic studies; or a CPG validated on a test set. | SR (with homogeneity*) of Level 1 economic studies |
| A | 1b | Individual RCT (with narrow Confidence Interval^iii^) | Individual inception cohort study with > 80% follow-up | Independent blind comparison of an appropriate spectrum of consecutive patients, all of whom have undergone both the diagnostic test and the reference standard. | Analysis comparing all (critically-validated) alternative outcomes against appropriate cost measurement, and including a sensitivity analysis incorporating clinically sensible variations in important variables. |
|  | 1c | All or none^iv^ | All or none case-series^v^ | Absolute SpPins and SnNouts^vi^ | Clearly as good or better,^vii^ but cheaper. Clearly as bad or worse but more expensive. Clearly better or worse at the same cost. |
|  | 2a | SR (with homogeneity*) of cohort studies | SR (with homogeneity*) of either retrospective cohort studies or untreated control groups in RCTs. | SR (with homogeneity*) of Level >2 diagnostic studies | SR (with homogeneity*) of Level >2 economic studies |
| B | 2b | Individual cohort study (including low quality RCT; e.g., <80% follow-up) | Retrospective cohort study or follow-up of untreated control patients in an RCT; or CPG not validated in a test set. | Any of:   - Independent blind or objective comparison; - Study performed in a set of non-consecutive patients, or confined to a narrow spectrum of study individuals (or both) all of whom have undergone both the diagnostic test and the reference standard; - A diagnostic CPG not validated in a test set. | Analysis comparing a limited number of alternative outcomes against appropriate cost measurement, and including a sensitivity analysis incorporating clinically sensible variations in important variables. |
|  | 2c | “Outcomes” Research | “Outcomes” Research |  |  |
|  | 3a | SR (with homogeneity*) of case-control studies |  |  |  |
|  | 3b | Individual Case-Control Study |  | Independent blind comparison of an appropriate spectrum, but the reference standard was not applied to all study patients | Analysis without accurate cost measurement, but including a sensitivity analysis incorporating clinically sensible variations in important variables. |
| C | 4 | Case-series (and poor quality cohort and case-control studies^viii^) | Case-series (and poor quality prognostic cohort studies^ix^) | Any of:   - Reference standard was unobjective, unblinded or not - independent; - Positive and negative tests were verified using separate reference standards; - Study was performed in an inappropriate spectrum** of patients. | Analysis with no sensitivity analysis |
| D | 5 | Expert opinion without explicit critical appraisal, or based on physiology, bench research or “first principles” | Expert opinion without explicit critical appraisal, or based on physiology, bench research or “first principles” | Expert opinion without explicit critical appraisal, or based on physiology, bench research or “first principles” | Expert opinion without explicit critical appraisal, or based on economic theory |

1. These levels were generated in a series of iterations among members of the NHS R&D Centre for Evidence-Based Medicine (Chris Ball, Dave Sackett, Bob Phillips, Brian Haynes, and Sharon Straus).
2. Recommendations based on this approach apply to “average” patients and may need to be modified in light of an individual patient’s unique biology (risk, responsiveness, etc.) and preferences about the care they receive.
3. Users can add a minus-sign “-“ to denote the level of that fails to provide a conclusive answer because of:

- EITHER a single result with a wide Confidence Interval (such that, for example, an ARR in an RCT is not statistically significant but whose confidence intervals fail to exclude clinically important benefit or harm)
- OR an SR with troublesome (and statistically significant) heterogeneity.
- Such evidence is inconclusive, and therefore can only generate Grade D recommendations.

1. By homogeneity we mean a systematic review that is free of worrisome variations (heterogeneity) in the directions and degrees of results between individual studies. Not all systematic reviews with statistically significant heterogeneity need be worrisome, and not all worrisome heterogeneity need be statistically significant. As noted above, studies displaying worrisome heterogeneity should be tagged with a “-“ at the end of their designated level.
2. Clinical Prediction Guide
3. See note #2 for advice on how to understand, rate and use trials or other studies with wide confidence intervals.
4. Met when all patients died before the Rx became available, but some now survive on it; or when some patients died before the Rx became available, but none now die on it.
5. Met when there are no reports of anyone with this condition ever avoiding (all) or suffering from (none) a particular outcome (such as death).
6. An “Absolute SpPin” is a diagnostic finding whose Specificity is so high that a Positive result rules-in the diagnosis. An “Absolute SnNout” is a diagnostic finding whose Sensitivity is so high that a Negative result rules-out the diagnosis.
7. Good, better, bad, and worse refer to the comparisons between treatments in terms of their clinical risks and benefits.
8. By poor quality cohort study we mean one that failed to clearly defined comparison groups and/or failed to measure exposures and outcomes in the same (preferably blinded), objective way in both exposed and non-exposed individuals and/or failed to identify or appropriately control known confounders and/or failed to carry out a sufficiently long and complete follow-up of patients. By poor quality case-control study we mean one that failed to clearly define comparison groups and/or failed to measure exposures and outcomes in the same blinded, objective way in both cases and controls and/or failed to identify or appropriately control known cofounders.
9. By poor quality prognostic cohort study we mean one in which sampling was biased in favour of patients who already had the target outcome, or the measurement of outcomes was accomplished in <80& of study patients, or outcomes were determined in an unblinded, non-objective way, or there was no correction for confounding factors.

Abbreviations:

SR – Systematic review

RCT – Randomised Controlled Clinical Trial

CPG – Clinical Prediction Guide

ARR – Absolute Risk Reduction

Rx - Prescription
