# Supplementary material for: Systems for grading the quality of evidence and the strength of recommendations I: Critical appraisal of existing approaches The GRADE Working Group
Source: BMC Health Serv Res. 2004 Dec 22;4:38. doi: 10.1186/1472-6963-4-38 (PMC545647; doi:10.1186/1472-6963-4-38)
Supplement: Additional File 4 — Scottish Intercollegiate Guidelines (SIGN), a brief description of the SIGN approach. [file 1472-6963-4-38-S4.doc]

**APPENDIX 4.**

**Scottish Intercollegiate Guidelines (SIGN)**

Brief description prepared by Bob Phillips.

**Background**

The Scottish Intercollegiate Guidelines Network was established in 1993 to develop evidence-based clinical guidelines for the National Health Service in Scotland. In the past, it has graded recommendations using the system developed by US AHCPR. From Autumn 2000 onwards, SIGN has used a revised methodology for *grading* guideline recommendations, which is under review here ([www.sign.ac.uk/methodology/](http://www.sign.ac.uk/methodology/)). The guidelines produced are intended for a wide range of healthcare professionals. They cover a breadth of clinical areas spanning surgical and medical topics and primary care and secondary care settings.

**Quality of evidence**

Quality of evidence is assessed by assigning a *level of evidence.* (Details can be found at [www.sign.ac.uk/guidelines/fulltext/50/index.html](http://www.sign.ac.uk/guidelines/fulltext/50/index.html)) The levels are arranged from 1++ (least likely to be biased) to 4 (greatest potential for bias), with 8 ranks. Studies are assessed on the basis of critically appraisal checklists. The checklists have been derived from the Method for evaluating research and guidelines evidence (MERGE) produced by the New South Wales Department of Health. They are intended to be completed by users from different health backgrounds and degrees of expertise. The study design and a qualitative assessment of answers to the checklist questions give the *level*. Different critical appraisal questions are intended to be used with different study types (diagnostic, therapeutic, etiologic etc) with cohort and case-control studies reviewed only by people with expertise in the field.

**Strength of recommendations**

Strength of recommendation is communicated by a scale from A to D. The *grade of recommendation* is drawn from the *level* of evidence and "considered clinical judgement". This includes the size and consistency of the body of evidence, its applicability, clinical impact (including economic factors) and generalisablity (which takes the values of the target population into account). The recommendation is then assigned a *grade*, but additionally the wording of the recommendation will reflect the strength of recommendation.

**Strengths and weaknesses**

The strengths of the new SIGN approach are its structural simplicity and potential to discriminate between different study design requirements for different clinical questions. The levels of evidence are likely to be reproducible. Its weaknesses include the unstructured formation of grades of recommendation. The definition of "considered judgement" outlines many areas to be considered. There is a clear explanation of how study quality may limit the grade of recommendation. Assimilation of the other factors is not well described. There is no way of assessing or challenging these considerations, and the method is unlikely to be reproducible.

**Target audiences**

The SIGN guidelines are intended for use by a wide audience of healthcare providers, including doctors, nurses, and managers.

**Guidelines made with the use of this approach**

49 guidelines have been completed (see [www.sign.ac.uk/guidelines/published/](http://www.sign.ac.uk/guidelines/published/)) in areas as diverse as dentistry, cancer care and child/adolescent mental health.

**Evaluations of the approach**

No formal evaluations appear to have been completed of the SIGN methodology. Informal evaluations report good acceptance of the documents as rigorous, authoritative and clinically useful.
