# Supplementary material for: Systems for grading the quality of evidence and the strength of recommendations I: Critical appraisal of existing approaches The GRADE Working Group
Source: BMC Health Serv Res. 2004 Dec 22;4:38. doi: 10.1186/1472-6963-4-38 (PMC545647; doi:10.1186/1472-6963-4-38)
Supplement: Additional File 5 — U.S. Preventive Services Task Force (USPSTF), a brief description of the USPSTF approach. [file 1472-6963-4-38-S5.doc]

**APPENDIX 5.**

**U.S. Preventive Services Task Force (USPSTF)**

Brief description prepared by David Atkins.

**Background**

The U.S. Preventive Services Task Force (USPSTF) was established in 1984 to systematically review the evidence of effectiveness of preventive services, including common screening tests, counselling , immunizations, and chemopreventive agents. The USPSTF, which is supported by but independent of the U.S. Department of Health and Human Services, built upon methodology developed by the Canadian Task Force on the Periodic Health Examination (CTFPHE) [1]. The first report of the USPSTF was published in 1989 as the *Guide to Clinical Preventive Services* [2]*,* and revised in 1996 [3]. A third USPSTF was appointed in 1998 to update the 1996 recommendations. The first four of these updates, including a description of the USPSTF history [4] and methodology [5] were published in April 2001 [6]. These materials, as well as access to the full text of the 1996 report, are available at the USPSTF Website [7].

**Levels of evidence**

The USPSTF has expanded on the hierarchy it originally adapted from the CTFPHE (see Table 1) to revise and make more explicit some aspects of its methodology. A key concept introduced by the second USPSTF was the use of “**causal pathways**” (now termed “**analytic frameworks**”) to identify discrete key questions that need to be answered to evaluate the benefits and harms of a given service (see Figure 1).

Quality of evidence is rated on 3 levels: the individual study, the body of evidence for each key question (“link” in analytic framework), and the collected evidence for the whole service. Individual studies are categorized by study design (Table 1). For each of 5 different study designs (systematic reviews, RCTs, case-control studies, cohort studies, and diagnostic accuracy studies), quality is then further classified based on criteria modified from the Evidence Based Medicine working group. In general, a “good” study meets all criteria for that study design; a "fair” study does not meet all criteria but is judged to have no “fatal flaw” that invalidates its results; and a “poor” study contains a fatal flaw (e.g., lack of intention to treat analysis, noncomparable groups, etc.

Body of evidence for each link is rated qualitatively on three criteria: internal validity, external validity, and “coherence” (i.e. consistency among studies and with other supporting evidence). Overall evidence is rated considering the directness of the available evidence and the quality of evidence for individual links in the analytic framework (Table 2).

**Strength of recommendations**

Strength of recommendation is rated on a scale of A to D (see Table 3 for wording). An I category designates “Insufficient Evidence” to recommend for or against. Ratings reflect two dimensions: quality of evidence and assessment of balance of harms and benefits.

| Quality of Overall Evidence of Effectiveness | Estimate of Net Benefit (Benefit Minus Harms) | | | |
| --- | --- | --- | --- | --- |
|  | Substantial | Moderate | Small | Zero/Negative |
| Good | A | B | C | D |
| Fair | B | B | C | D |
| Poor | I – Insufficient Evidence | | | |

**Strengths and weaknesses**

Strengths include:

- Analytic frameworks make explicit key questions and can be adapted to other treatment questions
- Clear and direct linkage between quality of evidence and strength of recommendation
- Recommendations using A-D letter grades communicates clearly to clinicians
- Considers other elements of evidence beyond study design.
- Weighs benefits and harms

Limitations and challenges include:

- The approach, with different levels of analysis, can seem complex
- Subjective judgements required in integrating different dimensions into an overall assessment of strength of evidence
- May not adapt as easily to prognostic/diagnostic questions
- Assessments don’t always adjust for individual patient values.
- Difficult to make recommendations in the absence of good evidence.

**Target audiences**

The primary target audience for USPSTF recommendations is primary care clinicians. Additional audiences who have used USPSTF recommendations include professional organizations, health plans, insurers, quality organizations, purchasers and policy makers.

There is no empirical data evaluating the level of understanding of USPSTF methods/recommendations, but awareness is highest among advanced practice nurses and family physicians and lowest among pediatricians and ob/gyns. We are planning an evaluation of the use of USPSTF materials in teaching programs and have done focus group studies with different clinical audiences.

**Guidelines**

USPSTF recommendations cover 70 specific topic areas and over 200 services. These recommendations have been adopted by many health plans, and have been adapted by the American Academy of Family Physicians and other organizations.

**References**

1. Canadian Task Force on the Periodic Health Examination. The periodic health examination. *Can Med Assoc J.* 1979;121:1194-254. ([http://www.ctfphc.org](http://www.ctfphc.org/))
2. U.S. Preventive Services Task Force. *Guide to Clinical Preventive Services: An Assessment of the Effectiveness of 169 Interventions.* Baltimore: Williams and Wilkins; 1989.
3. U.S. Preventive Services Task Force. *Guide to Clinical Preventive Services, 2^nd^ ed.* Baltimore, MD: Williams and Wilkins; 1996.
4. Atkins D, Best D, Shapiro EN. The third U.S. Preventive Services Task Force : background, methods and first recommendations. *Am J Preventive Medicine.* 2001;20:3 (supplement 1): 1-108.
5. Woolf SH, Atkins D. The evolving role of prevention in health care: Contributions of the U.S. Preventive Services Task Force. *Am J Preventive Medicine.* 2001;20:3 (supplement 1):13-20.
6. Harris RP, Helfand M, Woolf SH et al. for the Methods Work Group of the Third U.S. Preventive Services Task Force. Current methods of the U.S. Preventive Services Task Force: A review of the process. *Am J Preventive Medicine.* 2001;20:3 (Supplement 1) : 21-35.
7. <http://www.ahrq.gov/clinic/uspstfix.htm>

**Figure 1. USPSTF Analytic Frameworks (Example)**

1

2

3

Early detection of disease

4

5

Screen

Asymptomatic

patient

- avg. risk
- high risk

Treatment

Reduction in :

- morbidity
- mortality

**Table 1. Quality of Evidence for Individual studies: Study Design***.*

I At least one well-conducted RCT

II-1 Controlled trials without randomization

II-2 Well-designed cohort or case-control studies, preferably from multiple sites

II-3 Multiple time-series with or without intervention. Dramatic before-after results (e.g. penicillin)

III Expert opinion

**Table 2. Quality of Overall Evidence for an Intervention**

**The quality of the overall evidence for a service is classified on a three-point scale (good, fair, poor):**

**Good:** Consistent results from high quality studies in representative populations demonstrate direct and clinically important positive effects on the most important health outcomes.

**Fair:** Evidence is sufficient to demonstrate clinically important positive effects on health outcomes, but is limited by the number, quality or consistency of the individual studies, generalizability to routine practice, or directness of the evidence on most important outcomes.

**Poor:** Evidence is insufficient to demonstrate positive effects on health outcomes, due to limited number or power of studies, important flaws in design or conduct of studies, or lack of important health outcomes.

Table 3. U.S. Preventive Services Task Force Recommendations and Ratings

**Task Force recommendations are graded on a five-point scale ( A, B, C, D, I), reflecting the strength of evidence in support of the intervention:**

**A:** The USPSTF strongly recommends that these services be routinely provided to eligible patients. There is good evidence that the service improves health outcomes and the USPSTF concludes that benefits substantially outweigh harms.

**B:** The USPSTF recommends routinely providing the service to eligible patients. There is at least fair evidence that the service improves health outcomes and the USPSTF concludes that benefits outweigh harms.

**C:** The USPSTF makes no recommendation for or against regarding routine use of the service. There is fair or good evidence that the service can improve some health outcomes, but the USPSTF concludes the balance of benefits and harms is too close to make a general recommendation. The decision may be sensitive to individual patient preference.

**D:** The USPSTF recommends against routine use of the service in asymptomatic patients. There is fair or good evidence that the service is ineffective or that the harms outweigh the benefits.

**I:** The USPSTF concludes that the evidence is insufficient to recommend for or against routinely providing the service. Evidence that the service is effective is either of poor quality or is conflicting. Other factors may be considered in determining appropriate practice.
