# Supplementary material for: Systems for grading the quality of evidence and the strength of recommendations I: Critical appraisal of existing approaches The GRADE Working Group
Source: BMC Health Serv Res. 2004 Dec 22;4:38. doi: 10.1186/1472-6963-4-38 (PMC545647; doi:10.1186/1472-6963-4-38)
Supplement: Additional File 6 — U.S. Task Force on Community Preventive Services (USTFCPS), a brief description of the USTFCPS approach. [file 1472-6963-4-38-S6.doc]

**APPENDIX 6.**

**U.S. Task Force on Community Preventive Services (USTFCPS)**

Brief description prepared by Peter Briss.

**Background:**

The *Guide to Community Preventive Services* (*Community Guide*) is being developed by the independent non-federal Task Force on Community Preventive Services (Task Force) supported by the U.S. Centers for Disease Control and Prevention (CDC) and others [1]. The Community Guide provides systematic reviews and evidence-based recommendations about population-based interventions (e.g., mass media, behavior change, and environmental interventions). More information about the Community Guide is available at [http://www.thecommunityguide.org](http://www.thecommunityguide.org/).

Systematic reviews are important for providing timely and accurate scientific information to support decision making, and for reducing the impact of chance and bias on conclusions. Systematic reviews of evaluations of population-based interventions , however, differ in some ways from those of individually-oriented clinical care interventions. For example, in population-based research, the randomized controlled trial (RCT) is less central than it is in clinical research. In population-based research the RCT is not always ethical or feasible, may have limited internal validity (e.g., due to contamination or inability to control confounding variables because individual randomization is not possible), or may have serious threats to external validity.

**Quality of evidence**

Community Guide methods have been previously described in detail [2-4]. Briefly, diverse teams conduct the reviews. Individual studies of intervention effectiveness are identified, their quality assessed and their results extracted. The body of evidence for an intervention’s effectiveness is then characterized as strong, sufficient, or insufficient based on the number of available studies, the strength of their design and execution, and the size and consistency of reported effects (Table 1).

*Suitability of study design* for assessing effectiveness is based on characteristics that protect against various potential threats to validity. *Quality of study execution* is assessed based on: 1) study population and intervention descriptions, 2) sampling, 3) exposure and outcome measurement, 4) data analysis, 5) interpretation of results (including follow-up, bias, and confounding), and 6) any other characteristics that are not addressed elsewhere. Each study is categorized as having good, fair, or limited quality of execution based on the number of limitations noted (9 are possible). Studies with 0–1, 2–4, and 5 or more limitations are categorized as having good, fair, and limited execution, respectively. Studies with limited execution are not used to support recommendations. *Numbers of studies:* All other characteristics being comparable, more studies (i.e., more replications) constitute a stronger body of evidence. Sufficient or strong evidence can be based either on a small number of studies with better execution and more suitable design or a larger number of studies with less suitable design or weaker execution. *Consistency of results* is defined as being generally consistent in direction and size based on the opinion of the Task Force*. Effect sizes* are defined to be large, intermediate or small based on the opinion of the Task Force. In general, larger effect sizes (e.g., absolute or relative risks) are considered to represent stronger evidence of effectiveness than smaller effects. *Expert opinion* can be applied by the Task Force when other evidence is not available, but had not been used as of May, 2001.

# Strength of recommendations

In general, strength of evidence of effectiveness (Table 1) links directly to strength of recommendation. Evidence other than effectiveness[[1]](#footnote-2) rarely may be incorporated in Task Force recommendations. For example, an intervention with harms thought by the Task Force to be out of proportion to its benefits would not be recommended even if effective in improving some outcomes.

**Strengths and weaknesses**

This method used to develop the Community Guide has several important strengths. The contributions of people with a broad range of backgrounds and perspectives limit institutional and individual biases. The many kinds of evidence included (e.g. effectiveness, economic evaluations, etc.) provide important information to support decision making. The assessment of effectiveness that incorporates many different factors (e.g., study design, study execution, numbers of studies, etc.) allows a broad range of public health interventions to be evaluated in ways that incorporate both scientific rigor and the feasibility and appropriateness of the evaluation. A feasible, evidence-based approach to public health has been argued to be a positive development, bringing “public health to the same level of scientific scrutiny … [as evidence-based medicine]” [5].

Limitations of the approach include its complexity and cost in terms of time, resources, and expertise required, the possibility that some parts of the process could be seen as arbitrary (e.g., numbers of studies required), that aspects of the process that depend on Task Force opinion might not result in identical conclusions given a different group of decision makers, and that the recommendations unavoidably do not incorporate all of the information that will be important to policy makers.

**Target audiences**

The primary audience for Community Guide recommendations includes persons involved in planning, funding, and implementing population-based services and policies to improve health at the state and local levels in the U.S. These include federal agencies, state and local health departments, legislators, managed care, and purchasers of health care and public health services. Materials developed for the primary audiences should be useful for other audiences as well.

**Guidelines developed using this approach**

As of May, 2001, the Task Force has published 43 reviews and recommendations [6-13] with another 31 completed and awaiting publication. Several groups have adopted part or all of the Community Guide approach to reviewing evidence. We are not aware that other groups have used this approach to translate evidence into recommendations.

**Studies evaluating the application of guidelines developed using this approach**

Formal evaluations completed, underway, or planned: There are, as yet, no evaluations of the entire process. Evaluations of parts of the process, (e.g., inter-rater reliability of the assessment of study execution) are underway. Evaluations of the effects of the published reviews and recommendations on implementation decisions are also underway.

Informal evaluations: Focus group testing of the first published set of reviews and recommendations^6-9^ with audiences of people who implement relevant programs indicated a desire for this type of information and general satisfaction with the content.

**References**

1. Truman BI, Smith-Akin CK, Hinman AR, et al. Developing the Guide to Community Preventive Services--overview and rationale. The Task Force on Community Preventive Services. American Journal of Preventive Medicine 2000;18:18-26.

2. Briss PA, Zaza S, Pappaioanou M, et al. Developing an evidence-based Guide to Community Preventive Services--methods. The Task Force on Community Preventive Services. American Journal of Preventive Medicine 2000;18:35-43.

3. Zaza S, Wright-De A, Briss PA, et al. Data collection instrument and procedure for systematic reviews in the Guide to Community Preventive Services. Task Force on Community Preventive Services. American Journal of Preventive Medicine 2000;18:44-74.

4. Carande-Kulis VG, Maciosek MV, Briss PA, et al. Methods for systematic reviews of economic evaluations for the Guide to Community Preventive Services. Task Force on Community Preventive Services. [see comments]. American Journal of Preventive Medicine 2000;18:75-91.

5. McGinnis JM, Foege W. Guide to Community Preventive Services: harnessing the science. American Journal of Preventive Medicine 2000;18:1-2.

6. Briss PA, Rodewald LE, Hinman AR, et al. Reviews of evidence regarding interventions to improve vaccination coverage in children, adolescents, and adults. The Task Force on Community Preventive Services. [Review] [251 refs]. American Journal of Preventive Medicine 2000;18:97-140.

7. Shefer A, Briss P, Rodewald L, et al. Improving immunization coverage rates: an evidence-based review of the literature. [Review] [251 refs]. Epidemiologic Reviews 1999;21:96-142.

8. Vaccine-preventable diseases: improving vaccination coverage in children, adolescents, and adults. A report on recommendations from the Task Force on Community Preventive Services. MMWR - Morbidity & Mortality Weekly Report 1999;48:1-15.

9. Recommendations regarding interventions to improve vaccination coverage in children, adolescents, and adults. Task Force on Community Preventive Services. American Journal of Preventive Medicine 2000;18:92-6.

10. Hopkins DP, Briss PA, Ricard CJ, et al. Reviews of evidence regarding interventions to reduce tobacco use and exposure to environmental tobacco smoke. [Review] [429 refs]. American Journal of Preventive Medicine 2001;20:16-66.

11. Recommendations regarding interventions to reduce tobacco use and exposure to environmental tobacco smoke. American Journal of Preventive Medicine 2001;20:10-5.

1. Strategies for reducing exposure to environmental tobacco smoke, increasing tobacco-use cessation, and reducing initiation in communities and health-care systems. A report on recommendations of the Task Force on Community Preventive Services. MMWR - Morbidity & Mortality Weekly Report 2000;49:1-11.
2. Motor-vehicle occupant injury: Strategies for increasing use of child safety seats, increasing use of safety belts, and reducing alcohol-impaired driving. A report on recommendations of the Task Force on Community Preventive Services. MMWR - Morbidity & Mortality Weekly Report 2001;50:1-16.

| Table 1. Assessing the Strength of a Body of Evidence on Effectiveness of Population-Based Interventions in the Guide to Community Preventive Services. | | | | | | |
| --- | --- | --- | --- | --- | --- | --- |
| Evidence of Effectiveness^a^ | Execution C  Good or Fair^b^ | Design Suitability C  Greatest,  Moderate, or Least | Number  of Studies | Consistent^c^ | Effect  Size^d^ | Expert Opinion^e^ |
| *Strong* | Good | Greatest | At Least 2 | Yes | Sufficient | Not Used |
|  | Good | Greatest or Moderate | At Least 5 | Yes | Sufficient | Not Used |
|  | Good or Fair | Greatest | At Least 5 | Yes | Sufficient | Not Used |
|  | Meet Design, Execution, Number, and Consistency Criteria for Sufficient But Not Strong Evidence | | | | Large | Not Used |
| *Sufficient* | Good | Greatest | 1 | Not Applicable | Sufficient | Not Used |
|  | Good or Fair | Greatest or Moderate | At Least 3 | Yes | Sufficient | Not Used |
|  | Good or Fair | Greatest, Moderate, or Least | At Least 5 | Yes | Sufficient | Not Used |
| *Expert Opinion* | Varies | Varies | Varies | Varies | Sufficient | Supports a Recommendation |
| *Insufficient^f^* | A. Insufficient Designs or Execution | | B. Too Few Studies | C. Inconsistent | D. Small | E. Not Used |

**Notes to Table 1.**

^a^The categories are not mutually exclusive; a body of evidence meeting criteria for more than one of these should be categorized in the highest possible category.

^b^Studies with limited execution are not used to assess effectiveness.

^c^ Generally consistent in direction and size

^d^Sufficient and large effect sizes are defined on a case-by-case basis and are based on Task Force opinion.

^e^Expert opinion will not be routinely used in the *Guide* but can affect the classification of a body of evidence as shown.

^f^Reasons for a determination that evidence is insufficient will be described as follows: A. Insufficient designs or executions, B. Too few studies, C. Inconsistent. D. Effect size too small, E. Expert opinion not used. These categories are not mutually exclusive and one or more of these will occur when a body of evidence fails to meet the criteria for strong or sufficient evidence.

1. Community Guide reviews also include systematically collected and evaluated information on the applicability of effectiveness data (i.e., the extent to which available effectiveness data is thought to apply to additional populations and settings); the intervention’s other effects (i.e., side effects, including important outcomes of the intervention not already included in the assessment of effectiveness whether they are harms or benefits, intended or not intended, and health or non-health outcomes); economic impact; and barriers that have been observed when implementing interventions. In general this additional information does not directly influence the recommendations. [↑](#footnote-ref-2)
